# Retinal Nerve Fiber Layer Features Identified by Unsupervised Machine Learning on Optical Coherence Tomography Scans Predict Glaucoma Progression

**DOI:** 10.1167/iovs.17-23387

**Published:** 2018-06

**Authors:** Mark Christopher, Akram Belghith, Robert N. Weinreb, Christopher Bowd, Michael H. Goldbaum, Luke J. Saunders, Felipe A. Medeiros, Linda M. Zangwill

**Affiliations:** 1 Department of Ophthalmology, Hamilton Glaucoma Center, Shiley Eye Institute, University of California San Diego, La Jolla, California, United States; 2 Duke Eye Center, Department of Ophthalmology, Duke University, Durham, North Carolina, United States

**Keywords:** machine learning, glaucoma progression, retinal nerve fiber layer

## Abstract

**Purpose:**

To apply computational techniques to wide-angle swept-source optical coherence tomography (SS-OCT) images to identify novel, glaucoma-related structural features and improve detection of glaucoma and prediction of future glaucomatous progression.

**Methods:**

Wide-angle SS-OCT, OCT circumpapillary retinal nerve fiber layer (cpRNFL) circle scans spectral-domain (SD)-OCT, standard automated perimetry (SAP), and frequency doubling technology (FDT) visual field tests were completed every 3 months for 2 years from a cohort of 28 healthy participants (56 eyes) and 93 glaucoma participants (179 eyes). RNFL thickness maps were extracted from segmented SS-OCT images and an unsupervised machine learning approach based on principal component analysis (PCA) was used to identify novel structural features. Area under the receiver operating characteristic curve (AUC) was used to assess diagnostic accuracy of RNFL PCA for detecting glaucoma and progression compared to SAP, FDT, and cpRNFL measures.

**Results:**

The RNFL PCA features were significantly associated with mean deviation (MD) in both SAP (*R*^2^ = 0.49, *P* < 0.0001) and FDT visual field testing (*R*^2^ = 0.48, *P* < 0.0001), and with mean circumpapillary RNFL thickness (cpRNFLt) from SD-OCT (*R*^2^ = 0.58, *P* < 0.0001). The identified features outperformed each of these measures in detecting glaucoma with an AUC of 0.95 for RNFL PCA compared to an 0.90 for mean cpRNFLt (*P* = 0.09), 0.86 for SAP MD (*P* = 0.034), and 0.83 for FDT MD (*P* = 0.021). Accuracy in predicting progression was also significantly higher for RNFL PCA compared to SAP MD, FDT MD, and mean cpRNFLt (*P* = 0.046, *P* = 0.007, and *P* = 0.044, respectively).

**Conclusions:**

A computational approach can identify structural features that improve glaucoma detection and progression prediction.

With worsening of glaucoma damage, there are characteristic changes to the retinal nerve fiber layer (RNFL) in both the optic nerve head (ONH) and macula. For clinicians to detect such changes early, it is important to have sensitive and specific structural measurements.

Over the past decade, standard clinical management of glaucoma has incorporated imaging using spectral domain optical coherence tomography (SD-OCT) to assess both the ONH and macula. This imaging technology allows for direct observation of 3D retinal structure and objective, quantitative measurements of these regions. SD-OCT measurements have been studied extensively and shown to reliably detect and monitor glaucoma.^1–3^ ONH and peripapillary measurements including global and sectoral RNFL thickness (Hammel N, et al. *IOVS* 2015;56:ARVO E-Abstract 4568).^4^ minimum rim width,^5–7^ and lamina depth^8,9^ have been identified as predictors that can aid early detection and monitoring. Structural changes in the macula region, including thinning of the RNFL as well as ganglion cell and inner plexiform layers, have been associated with glaucomatous damage.^10,11^

More recently, swept source (SS)-OCT has become available as an alternative to standard SD-OCT. This imaging technique has a fast acquisition speed (∼100,000 A-scans per second) and can capture wide-angle scans that include both the ONH and macula regions.^12,13^ Previous work has shown SS-OCT measurements of RNFL thickness in both the ONH and macula regions are comparable to measurements derived from SD-OCT for diagnosing glaucoma.^14^ Unlike SD-OCT, these wide-angle images provide an opportunity to identify structural changes to ONH and macula regions simultaneously. These complex changes may not be well described by the common measurements that include global and sectoral averages of RNFL and ganglion cell layer (GCL) thickness. These standard thickness measurements attempt to represent the high-dimensional, complex RNFL structure using only a limited number of averaged thickness values. Applying data-driven techniques that learn a representation of RNFL structure may reveal latent features useful in characterizing and predicting disease. One such approach employs unsupervised machine learning to identify patterns in SS-OCT measurements of RNFL thickness. Broadly, unsupervised machine learning techniques can identify latent structure within high-dimensional data.^15^

Previous work has applied unsupervised techniques to identity patterns in visual field data that are related to glaucoma and glaucoma progression.^16–18^ A similar unsupervised approach has also been applied to identify ONH structural features using stereo fundus photographs.^19^ Here, we adapt and extend these approaches to identify novel RNFL structural features from a large SS-OCT dataset. These features can then be evaluated based on their ability to predict glaucomatous progression.

The aim of this report is to apply computational techniques to a large set of wide-angle SS-OCT images to identify novel, glaucoma-related structural features to improve detection of primary open angle glaucoma (POAG) and prediction of glaucomatous progression.

## Methods

### Participant Cohort

Participants included in this retrospective analysis of structural and functional data were originally were originally recruited from an existing cohort enrolled in the Diagnostic Innovations in Glaucoma (DIGS). DIGS is a longitudinal study designed to investigate structural and functional changes in glaucoma. Recruitment and methodology was approved by the University of California, San Diego (UCSD) Institutional Review Board. Recruitment and methodologies adhered to the tenets of the Declaration of Helsinki and were approved by the UCSD Institutional Review Board.

For inclusion in the cohort, participants were required to have no history of secondary glaucoma or other ocular diseases, intraocular surgery, stroke, Alzheimer, dementia, or life-threatening diseases. Participants were also required to have a baseline corrected visual acuity of 20/40 or better and less than 5.0 diopters spherical and 3.0 cylindrical refraction. At enrollment, measurements of intraocular pressure (IOP), central corneal thickness (CCT), and drainage angle were collected and standard automated perimetry (SAP) was performed. Participant eyes were examined using stereoscopic fundoscopy and simultaneous stereo photographs (Nidek/Topcon, Tokyo, Japan) were collected. Photographs were reviewed by two independent, masked graders and assigned grades of normal or glaucomatous. In the case of disagreement, a third experienced grader was consulted.

Participants were assigned to the glaucoma group if their photos indicated glaucomatous damage as judged by at least two experienced graders. Healthy participants were required to have normal optic disc photographs as well as an IOP <22 mm Hg and repeatable, normal SAP test results. RNFL measurements from OCT were not used to assign participants into the healthy or glaucoma groups. The total cohort consisted of 179 eyes of 93 glaucomatous participants and 56 eyes of 28 heathy participants. Data was collected at the baseline visit and at follow-up visits conducted every 3 months for a period of 2 years. See Table 1 for a summary of data collected at baseline and follow-up visits.

**Table 1:**
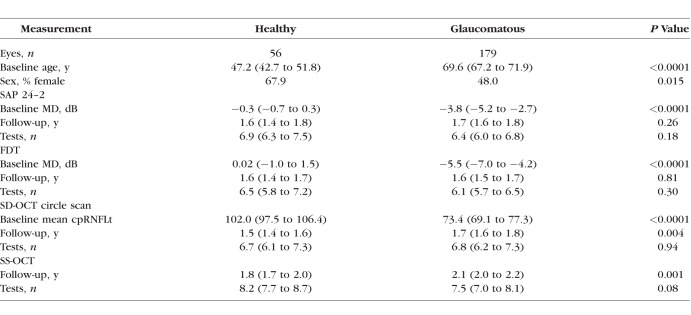
Summary of Baseline Characteristics and Follow-Up Data Collected

### Visual Field Testing

Visual field testing was performed at all visits using both SAP and frequency doubling technology (FDT) procedures. SAP testing was performed using the Humphrey Field Analyzer II (Carl Zeiss Meditec, Dubin, CA, USA) standard 24-2 testing pattern using the Swedish interactive thresholding algorithm. FDT testing was performed using the Humphrey Matrix (Carl-Zeiss Meditec). Tests that had more than 33% fixation losses, 33% false negative errors, or 15% false positive errors were exclude. Mean deviation (MD) and pattern standard deviation (PSD) were computed at baseline and each follow-up visit for subsequent analysis. Visual fields were processed and evaluated for quality according to standard protocols by the UCSD Visual Field Assessment Center.^20^

### OCT Imaging

At baseline and each follow-up visit, participants were imaged with SD-OCT (Spectralis; Heidelberg Engineering, Inc., Heidelberg, Germany) and deep range imaging SS-OCT (Triton; Topcon Medical Systems, Inc., Tokyo, Japan). Spectralis SD-OCT scans were acquired using the high resolution RNFL circle scan protocol that captured 1536 A-scans around the clinically standard 3.45 mm circle centered on the ONH.^21^ Images were processed and segmentation was performed using the built-in software (Spectralis version 5.4.7; Heidelberg Engineering, Inc.). The mean circumpapillary RNFL thickness (cpRNFLt) was automatically computed for each scan by averaging the segmented RNFL thickness around the circle scan. SS-OCT imaging was performed using a wide-angle protocol that captured a 12.0 × 9.0 mm scan including both the ONH and foveal regions. The RNFL thickness across this region (RNFL thickness map) was extracted for each scan using the built-in segmentation software (version 1.57). In both cases, scans were manually reviewed and those with imaging artifact, misalignment of the ONH and/or fovea, or segmentation errors were excluded by experienced graders following standard Imaging Data Evaluation and Analysis (IDEA) reading center protocols.^22^

### RNFL Map Feature Identification

Using the entire set of RNFL thickness maps extracted from all participant SS-OCT scans, structural RNFL features were identified using principal component analysis (PCA). PCA is a widely used technique for dimensionality reduction that takes advantage of correlations within image data to generate a new set of orthogonal features that explain major modes of variation observed within the input data. Each RNFL thickness map can then be represented as a weighted average of these new features with the weight magnitude indication the amount each feature contributes to a given RNFL thickness map. Previously, this approach has been used to identify features of ONH structure and predict glaucomatous damage.^19^

Prior to application of PCA, all SS-OCT scans from left eyes were flipped to right eye orientation. Scans were then registered within and across eyes. Across eyes, alignment was performed manually by a marking the location of the fovea and ONH in the baseline image of each eye. All baseline images were translated, rotated, and scaled so that fovea and ONH locations were aligned to an arbitrarily selected reference scan. Within each eye, follow-up scans were automatically aligned to its corresponding baseline scan. Automatic image registration was performed by applying an OpenCV implementation of the enhanced correlation coefficient algorithm to the thickness maps.^23,24^ Finally, RNFL thickness map values were standardized across scans so that each pixel location had a zero mean and unit variance across all thickness maps.

### Association Testing

Evaluation of the RNFL PCA features was performed first by testing for associations with other glaucoma-related measurements. These included both quantitative and categorical measurements collected at the baseline visit. Testing was performed to identify significant associations between RNFL PCA features and demographic variables (disease status and sex), functional measurements (SAP and FDT MD/PSD), structural measurements (mean cpRNFLt), and other clinical variables (IOP, CCT, visual acuity). For quantitative measurements, univariate linear regression models with RNFL PCA features as predictor variables were used. A significant regression slope between an RNFL PCA feature and another measurement indicated a statistically significant association. For categorical measurements, single-factor ANOVA tests were used to identify significant differences in RNFL PCA features across categories. Because of the significant difference in age between the glaucoma and healthy group (see Table 1), age was controlled for by including it as a covariate in all models. To determine significance, a *P* value threshold of 0.05 was selected and Bonferroni correction was used to account for multiple hypothesis testing.

### Glaucoma Detection

The accuracy of RNFL PCA features in detecting glaucoma was compared to the accuracy of standard clinical measurements. Specifically, RNFL PCA features were used to distinguish thickness maps of healthy eyes from those of glaucoma eyes. A logistic regression model predicting the probability of a thickness map coming from a glaucoma eye using the first 10 RNFL PCA features was constructed. This prediction was compared to predictions based on mean cpRNFLt from SD-OCT circle scans, mean deviation from 24-2 SAP (SAP MD), and mean deviation from FDT (FDT MD) captured within 30 days of the thickness map. Quantitative comparisons were performed using area under receiver operating characteristic (AUC). In building the predictive models, a participant-based “leave-one-out” approach was adopted to ensure separation of the training and test sets. Specifically, all scans from a participant were removed from the data set before PCA features and prediction models were trained on the rest of the data. The resulting model was then applied to the scans from the excluded participant. This was repeated for each participant to generate predictions for all scans. AUC values were estimated using a bootstrapping method that controlled for age.^25^

### Predicting Progression

Glaucoma eyes were classified into progressing or stable based on longitudinal measurements of mean cpRNFLt, SAP MD, and FDT MD collected during the 2-year follow-up period. This classification was performed based on criteria defined using a mixed effects modeling approach described previously.^26^ Mixed effects models are commonly used to help account for a lack of independence in a dataset. This can be a result of having repeated data points from the same individual (longitudinal measurements) or collecting data from related sources (measurements from both eyes of each participant). In this analysis, a separate mixed effects model was constructed for each measurement (mean cpRNFLt, SAP MD, FDT MD) that included per participant and per eye effects.^27^ A distribution of rates of change for each measurement was then computed using only the longitudinal data collected from healthy eyes. For a given measurement, the distribution of healthy rates was used to define criteria to classify glaucoma eyes into “progressing” and “stable” groups. Specifically, for each measurement, the rate of change of a glaucoma eye was estimated using linear regression and if that rate was faster than the 95th percentile of healthy eyes and significantly (*P* < 0.05) different than zero, it was considered a progressing eye. If the rate did not meet these criteria, the eye was considered stable. This provided a ground truth set of progressing and stable glaucoma eyes for each measurement (mean cpRNFLt, SAP MD, and FDT MD) that was used to evaluate the ability of RNFL PCA features to predict progression.

RNFL PCA features and standard clinical measurements were then evaluated based on their ability to distinguish progressing from stable glaucoma eyes. These evaluations were performed using only baseline data. For progression defined using each clinical measurement (mean cpRNFLt, SAP MD, and FDT MD), the AUC of baseline measurements in predicting progression was determined. Logistic regression models were used to compare the predictive ability of the first 10 RNFL PCA features and standard clinical measures to predict progression. As in the case of glaucoma detection, comparisons were performed using a leave-one-out strategy and age was controlled for when computing AUC.

## Results

### RNFL PCA Features

**Figure 1:**
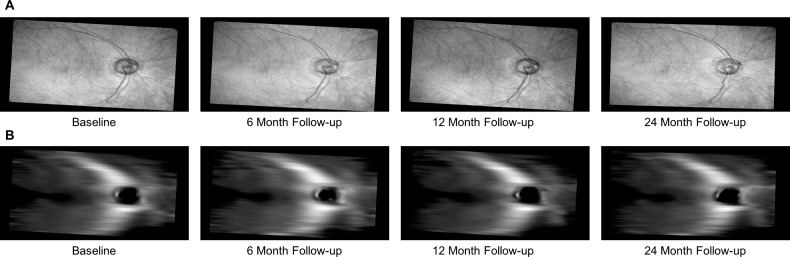
Example (A) en face and (B) RNFL thickness map images extracted longitudinally from a single glaucoma eye. These thickness maps were computed as the difference in depth between the segmented inner limiting membrane and nerve fiber layer. They served as input to the PCA-based approach to identify RNFL structural features.

Longitudinal RNFL thickness maps were extracted from wide-angle SS-OCT volumes of the 179 glaucomatous and 56 healthy eyes considered here. Figure 1 shows a set of RNFL en face and thickness maps at one point in time after registration for an example participant.

**Figure 2:**
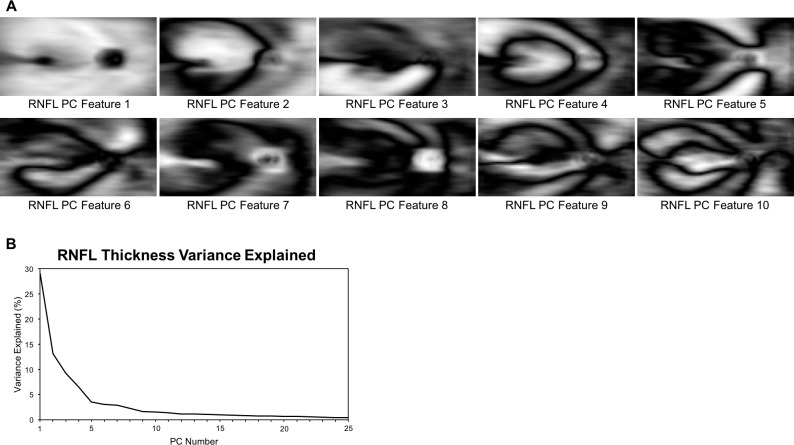
(A) Illustrations of the first 10 RNFL PCA features that were used for the analyses described here. Bright regions indicate areas where the feature affects RNFL thickness. (B) The proportion of variance in RNFL thickness maps explained by each feature. Cumulatively, the first 10 explained 75% of the variance.

**Figure 3:**
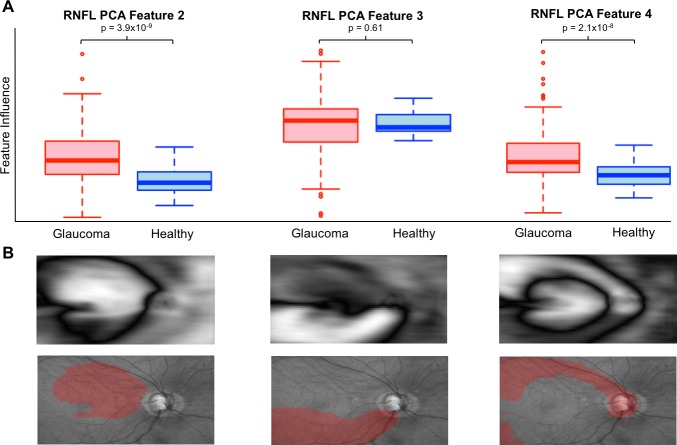
Illustration of the relationship between 3 example RNFL PCA features with (A) disease and (B) RNFL thinning. (A) Box plots showing the distribution of RNFL PCA features for glaucoma (red) and healthy eyes (blue). (B) RNFL PCA features (top) and the areas of greatest RNFL thinning associated with each overlaid on en face images (bottom, in red). RNFL PCA feature 2 shows a broad area of RNFL thinning that includes nasal and superior regions surrounding the macula. RNFL PCA features 3 and 4 identify thinning of superior and superior arcuate nerve fiber bundles.

The entire set of registered thickness maps were used as input to identify RNFL PCA features. These features are shown in Figure 2 along with the amount of variance explained by each. The choice of number of PCs to retain can be arbitrary. Here, 10 PCs were retained and used in all further analyses based on the amount of variance explained. These first 10 features explained ∼75% of the observed variance and additional PCs did little to increase predictive power. Each PC after 10 explained ∼1% or less of the variance in RNFL thickness map data. To aid in qualitative assessment of these features, Figure 3 illustrates the areas of greatest change in RNFL thickness associated with three examples of RNFL PCA features. RNFL PCA feature 2 shows clear change in the superior cpRNFL and macular area. RNFL PCA feature 3 is associated with RNFL thinning in an inferior arcuate pattern. RNFL PCA feature 4 indicates similar RNFL thinning but in a superior arcuate pattern.

### Feature Associations

**Figure 4:**
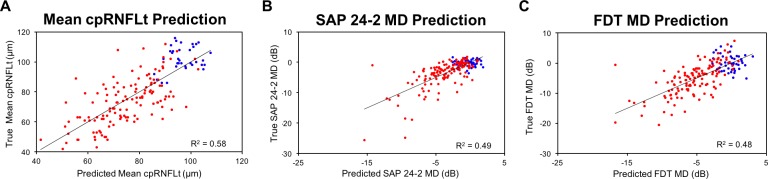
Scatterplots showing values predicted using RNFL PCA features versus true measurements for (A) mean cpRNFLt, (B) SAP 24-2 MD, and (C) FDT MD. RNFL PCA features were able to explain 58%, 49%, and 48% of the variance in these measurements, respectively. Heathy eyes are shown in blue and glaucoma eyes in red.

After Bonferroni correction, significant associations were identified between structure (as measured by RNFL PCA features) and a number of clinical measurements. Specifically, multivariate linear regression models identified significant associations with SAP MD (*R*^2^ = 0.49, *P* < 0.0001) and PSD (*R*^2^ = 0.52, *P* < 0.0001), FDT MD (*R*^2^ = 0.48, *P* < 0.0001) and PSD (*R*^2^ = 0.55, *P* < 0.0001), and mean cpRNFLt (*R*^2^ = 0.58, *P* < 0.0001). A multivariate logistic regression model identified significant associations with glaucoma status (*P* < 0.0001) were found. Table 2 summarizes these significant associations by RNFL PCA feature. Table 2 summarizes the significant associations between RNFL PCA features and clinical measurements. Figure 4 illustrates in scatterplots that mean cpRNFLt, SAP MD, and FDT MD values can be predicted from RNFL PCA features with *R*^2^ values of 58%, 49%, and 48%, respectively.

**Table 2:**
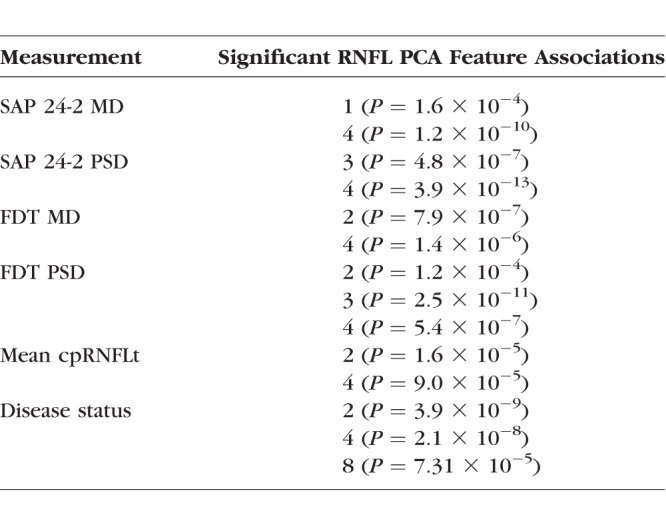
Significant Associations Between Clinical Measurements and RNFL PCA Features Tested Using Linear Regression for Quantitative Variables and ANOVA for Disease Status

### Glaucoma Detection

**Figure 5:**
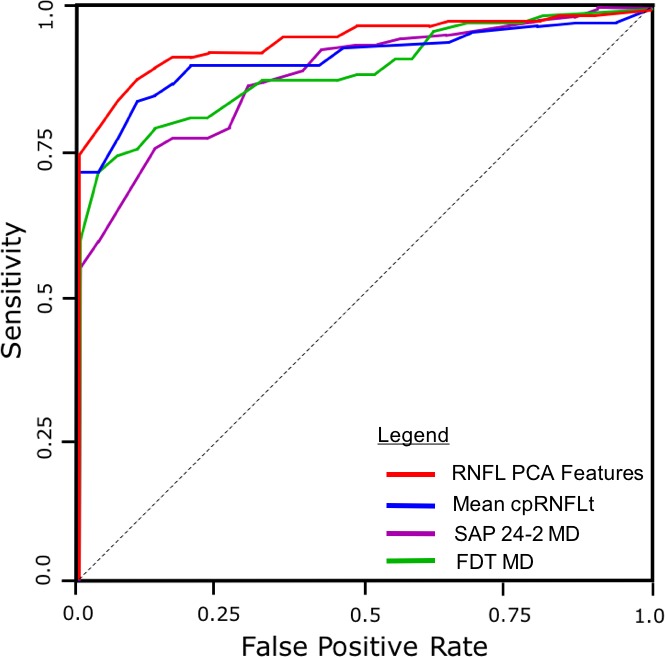
Receiver operating characteristic curves comparing performance in glaucoma detection between RNFL PCA features (AUC = 0.95), mean cpRNFLt (AUC = 0.90), SAP 24–2 MD (0.86), and FDT MD (AUC = 0.83).

The ability of the RNFL PCA features to detect glaucoma was compared to mean cpRNFLt, SAP MD, and FDT MD was evaluated using AUC (Fig. 5). The RNFL PCA features achieved the highest AUC of 0.95, followed by mean cpRNFLt (AUC = 0.90), SAP MD (AUC = 0.86), and FDT MD (AUC = 0.83). RNFL PCA feature performance was significantly higher than both SAP MD (*P* = 0.034) and FDT MD (*P* = 0.021). Table 3 summarizes glaucoma detection performance.

**Table 3:**
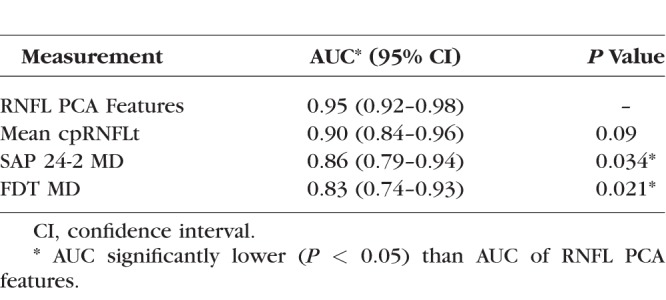
Diagnostic Accuracy for Glaucoma Detection by RNFL PCA Features and Clinical Measurements

### Predicting Progression

Progression was defined using a mixed-model based approach to identify glaucoma eyes that were changing faster than the 95th percentile of healthy eyes. Mean cpRNFLt resulted in 22 out of 179 (12.3%) of glaucoma eyes defined as progressing during the follow-up period. SAP MD resulted in detection of 16 (8.9%) progressing glaucoma eyes and FDT MD resulted in 13 (7.3%) progressing glaucoma eyes.

Clinical measurements and RNFL PCA features were evaluated based on their ability to predict progression using only baseline data. RNFL PCA features outperformed mean cpRNFLt (AUC 0.74 vs. 0.55, *P* = 0.044), SAP MD (AUC 0.74 vs. 0.58, *P* = 0.046), and FDT MD (AUC 0.71 vs. 0.52, *P* = 0.007) for predicting progression based on the baseline data. Table 4 summarizes progression prediction performance.

**Table 4:**
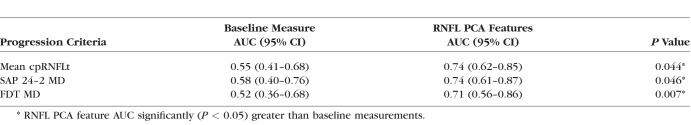
Progression Prediction of RNFL PCA Features is Significantly Better Than Standard Structural and Functional Measurements

## Discussion

A PCA-based approach was applied to a large SS-OCT dataset to identify novel features of RNFL structure. This approach identified objective, quantitative structural measurements of the RNFL that can simultaneously capture information about the ONH and macula regions. The resulting RNFL features were associated with standard structural and functional measurements as well as glaucoma. These features also improved diagnostic accuracy for POAG and predictions of glaucomatous progression compared to standard clinical measurements.

Previous work has evaluated numerous summary measurements derived from both SD-OCT and SS-OCT imaging for diagnosing POAG and predicting progression. Depending on the layer (e.g., RNFL versus GCIPL), region (e.g., macula versus ONH), and sector (e.g., superior versus inferior), these measurements can achieve high accuracy (AUC >0.9) in diagnosing POAG.^28^ Recent work has also suggested that measurements of different layers or regions may be most useful for monitoring structural changes at different stages of disease.^10,29–31^ One weakness of these metrics, however, is that they each measure only a single aspect of retinal structure. In addition, although the choice of what specific structures to measure is inspired by anatomic knowledge and clinical experience, the resulting set of measurements is not necessarily the best set for either detecting glaucoma or predicting disease progression. For this work, we adopted an approach that was unbiased approach with respect to changes in RNFL thickness—no prior information regarding areas of interest or specific types of RNFL changes were included in the models. Using unsupervised machine learning strategies, structural features important in POAG were identified solely on their ability to explain variance observed in the data. This type of approach can help supplement work where measurements are defined a priori by allowing the discovery of novel structural features that contribute to disease. The use of wide-angle SS-OCT imaging also allowed us to identify features that influence both macula and ONH structure simultaneously.

Previous work, by our group and others, has been successful in applying these unsupervised approaches to identify novel features useful for predicting progression in both structural and functional data. Unsupervised techniques able to identify both known patterns and novel patterns of visual field loss without knowledge of glaucoma status have been developed.^18,32^ These approaches were extended to aid in identifying progression in visual field loss and were able to outperform standard clinical metrics.^17^ Christopher et al.^19^ applied a PCA-based approach similar to the methods described here to structural measurements derived from stereo fundus photos and were also able improve glaucoma predictions beyond standard clinical measurements. These previous and current results suggest that use of unbiased, data-driven techniques may help reveal additional relationships between structure, function, and disease progression.

Identifying the areas of greatest change in RNFL thickness associated with each RNFL PCA feature can help evaluate the features qualitatively. The three features highlighted in Figure 3, for example, largely capture structural changes that could be relevant to the development and progression of POAG. RNFL PCA feature 2 is associated with a broad area of RNFL thinning that includes nasal and superior regions surrounding the macula. RNLF thinning in these regions has been shown to be predictive of POAG development.^11,33,34^ RNFL PCA feature 3 and 4 seem to measure thinning of inferior and superior arcuate nerve fiber bundles, respectively. Arcuate RNFL thinning in both sectors has been previously associated with POAG damage.^35,36^ These patterns also correspond to regions described by Hood et al.^37,38^ including inferior and superior arcuate loss (RNFL PCA features 3 and 4) as well as the region that covers the superior macula and temporal disc (RNFL PCA feature 2). In the current results, RNFL PCA feature 4 (superior arcuate thinning) is also strongly associated with visual field damage, cpRNFLt loss, and POAG (Table 2). The methods presented here were able to discover structural features that have been previously associated with POAG as well as identify new structural features that may help identify as-yet-unknown structural relationships.

The approaches and features described here may prove to be useful clinical tools, given the importance of early detection and early identification of progression in POAG. Failure to detect POAG or POAG progression is serious problem for clinicians and can lead to irreversible vison loss.^39,40^ RNFL PCA features increase diagnostic accuracy beyond that of standard clinical measurements suggesting a use in POAG screening or other early detection programs. With respect to POAG management, these features improve the ability to predict future functional and structural changes and could help guide care to prevent this progression. Qualitative assessment of these features also suggests that they represent distinct types RNFL loss and progression. Individual features may be useful in identifying patients with specific types of progression (e.g., RNFL PCA feature 4 measures superior arcuate loss, feature 2 measures diffuse thinning in superior macula and disc regions). Additional work with these features may help to identify different progression trajectories and aid clinicians in tailoring patient care.

There are a few limitations to the work presented here. First, there is a large difference in age between the healthy and glaucoma groups. Because retinal structure changes during aging, this difference could serve as a confounder for the analyses. This limitation was addressed by attempting to control for age by adding it as a covariate when appropriate. An additional issue with the cohort is the ratio of glaucoma to healthy participants in the sample under consideration here. Approximately three-quarters of the SS-OCT imaging data used to identify RNFL PCA features was collected from glaucoma participants. This is much a greater glaucoma proportion than general and at-risk populations and could limit the usefulness of features identified here. We addressed this issue by evaluating the glaucoma detection of RNFL PCA features on data that had been randomly resampled with at varying proportions of glaucoma. Using glaucoma proportions of 1%, 5%, 10%, and 25%, the glaucoma detection AUC of the features was evaluated as described in the methods. The AUC varied between 0.95 and 0.97 and similar to the AUC of 0.95, achieved on the nonresampled dataset. Another limitation is that only RNFL thickness was considered here. While the RNFL is an important structure in detecting and monitoring POAG, more recent work has shown that measurements of ganglion cell and inner plexiform layer (GCIPL) thickness could have comparable diagnostic accuracy and GCIPL thickness may be especially important in the macula.^11,41,42^ Because the SS-OCT imaging performed here included the macula as well as the ONH, including GCIPL thickness maps in future work may help improve predictive accuracy. Finally, while the PCA-based model used here is unbiased and relatively simple, it does not incorporate information about POAG status or functional measurements. This may limit its power to identify relationships between structure, function, and outcomes. Building models that explicitly maximize the ability of structural measures (e.g., thickness maps) to explain function and POAG status may reveal additional latent relationships and further improve predictions.

In summary, the work presented here applied unsupervised data-driven techniques to a large SS-OCT dataset to identify novel features of retinal structure that had better diagnostic accuracy than standard structural and functional measurements. These features were used as objective, quantitative measurements of RNFL structure covering a large region encompassing both the macula and ONH. They also improved accuracy in POAG diagnosis and predicting progression. Applied to additional datasets, this unbiased approach may help uncover other unknown structural relationships in glaucoma.
